# Three-dimensional deep learning to automatically generate cranial implant geometry

**DOI:** 10.1038/s41598-022-06606-9

**Published:** 2022-02-17

**Authors:** Chieh-Tsai Wu, Yao-Hung Yang, Yau-Zen Chang

**Affiliations:** 1 Department of Neurosurgery, Chang Gung Memorial Hospital, Taoyuan, 33305 Taiwan; 2 Department of Mechanical Engineering, Chang Gung University, Taoyuan, 33302 Taiwan

**Keywords:** Three-dimensional imaging, Machine learning, Computational science, Fracture repair

## Abstract

We present a 3D deep learning framework that can generate a complete cranial model using a defective one. The Boolean subtraction between these two models generates the geometry of the implant required for surgical reconstruction. There is little or no need for post-processing to eliminate noise in the implant model generated by the proposed approach. The framework can be used to meet the repair needs of cranial imperfections caused by trauma, congenital defects, plastic surgery, or tumor resection. Traditional implant design methods for skull reconstruction rely on the mirror operation. However, these approaches have great limitations when the defect crosses the plane of symmetry or the patient's skull is asymmetrical. The proposed deep learning framework is based on an enhanced three-dimensional autoencoder. Each training sample for the framework is a pair consisting of a cranial model converted from CT images and a corresponding model with simulated defects on it. Our approach can learn the spatial distribution of the upper part of normal cranial bones and use flawed cranial data to predict its complete geometry. Empirical research on simulated defects and actual clinical applications shows that our framework can meet most of the requirements of cranioplasty.

## Introduction

Cranioplasty^1,2^ is a surgical procedure in which cranial implants, or prostheses, are used to repair skull defects caused by trauma, congenital defects, plastic surgery, or tumor resection. The cranial implants must have an appropriate convex shape and fit accurately to the boundary of the defect. Their design usually involves time-consuming human–computer interaction using specific software and requires expertise in the medical field. For instance, Chen et al.^3^ utilized the geometry information of the mirrored model as the base to generate the implant model.

Considering that cranial defects may cross the plane of symmetry, and human cranial bones are usually asymmetrical, it is impractical to use the mirroring operation to generate the implant geometry. Therefore, there is a great need for automatic design of cranial implants.

In recent years, there have been substantial progresses in the image inpainting technology based on deep learning^4^. Image inpainting is the process of completing or repairing missing areas in a two-dimensional image. For example, Yan et al.^5^ introduced a shift-connection layer to the U-Net architecture^6^ for image completion that exhibits fast speed with promising fine details. Liao et al.^7^ proposed a deep convolutional neural networks scheme to explicitly separate content and style that generates fine-detailed and perceptually realistic inpainting results for structural and natural images. Besides, Pathak et al.^8^ combined the autoencoder network model^9–11^ with Generative Adversarial Network^12^ (GAN) to repair images and found that, in addition to reconstruction loss, an adversarial loss is beneficial in producing clear results. The schemes of Iizuka et al.^13^, Wang et al.^14^ and Jiang et al.^15^ are all based on the combination of autoencoder model^9–11^ and GAN^12^, in which a global context identifier and a local context identifier are used.

Compared with 2D images, 3D geometric models require more computing power to process^16,17^. In the inpainting of 3D models, the neural network architecture of Han et al.^18^ is divided into two parts, where the “Global Structure Inference” is responsible for the restoration of 32 × 32 × 32 low-resolution data, and the “Local Structure Refinement” part is responsible for refinement. Wang et al.^19^ also used GAN^12^ to train an Encoder–Decoder network^9–11^ to repair defects in 3D images with a resolution of 32 × 32 × 32 voxels. Dai et al.^20^ used a 3D encoder-predictor network to repair the defects of 3D images with a volumetric resolution of 32 × 32 × 32. The images are then replaced by higher resolution data by direct search. In addition, Wang et al.^21^ proposed a scheme that contains a local GAN^12^ and a global GAN^12^ to repair 3D mesh model in 80 × 80 × 80 voxels. The performance demonstrations of these contributions, however, are all based on simple geometric shapes such as airplanes, desks, and chairs.

Recently, Morais et al.^22^ proposed a deep learning approach, called Volumetric Convolutional Denoising Autoencoder, to perform 3D shape completion on defected skull models. This approach was evaluated on a full-skull reconstruction task and no verification of the generated implant geometry was provided. The deep learning approach of Li et al.^23^ is carried out in two steps using two neural networks. First, a network is trained to reconstruct the low-resolution version to locate the defective area. Second, another neural network is trained to make detailed implant predictions.

In addition, Shi and Chen^24^ proposed a convolutional neural network of the autoencoder^9–11^ structure with an auxiliary path to predict the 3D implant from inpainting 2D slices of different axes. Matzkin et al.^25^ used a 3D version of the standard U-Net architecture^6^ to compare two different approaches: direct estimation of the implant, and the reconstruct-and-subtract strategy, where the complete skull is first reconstructed, and then the defective model is subtracted from it to generate the implant. Before training, all the images were registered to an atlas space which is constructed by averaging several healthy head CT images. They concluded that the latter tends to generate noise in the implant models. In the succeeding work of Matzkin et al.^26^, an approximate shape prior, which is constructed by averaging several healthy head CT images, is concatenated with the input model to provide supplementary context information to the network. This modification is reported to facilitate the robustness of the model for out-of-distribution cases.

Nevertheless, these skull repair techniques are limited in feasible resolution, and the defects are all regular shapes produced by spherical or cubic masks. These shortcomings reduce its applicability in clinical practice.

The purpose of this research is to develop practical 3D inpainting techniques to automatically generate the geometry of the cranial implant, thereby eliminating subjectivity.

**Figure 1:**
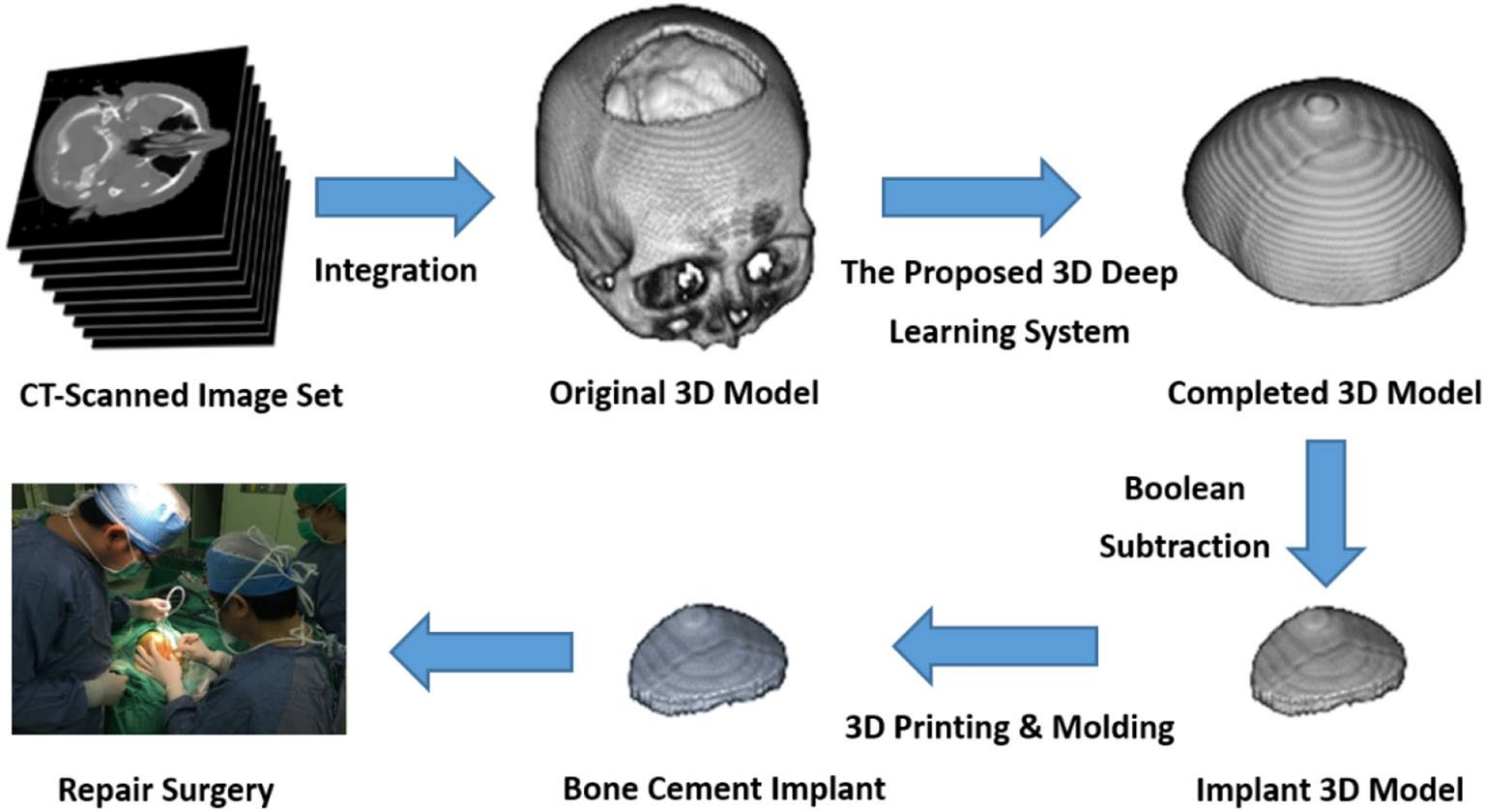
Cranial reconstruction surgery flowchart.

As shown in Fig. 1, the proposed cranioplasty procedure begins with integrating a defective 3D skull model using a CT-scanned image dataset. A completed cranial model is then automatically created by the proposed deep learning system. To reduce the computational burden, this study reduces the resolution of the 3D model and only generates the upper part of the cranium with a volumetric resolution of 112 × 112 × 40.

After that, an implant model is obtained by subtracting the defective model from the completed model. Subsequently, a template is made using 3D printing technology. The molding process is then applied to create the implant required for the repair surgery, which is made of bone cement in our surgical implementation.

For clinical practice, we resample and smooth the completed implant model to a volumetric resolution of 448 × 448 × 40. By subtracting the original defective model from the model again to remove residual voxels, a sufficiently smooth implant model can be obtained for 3D printing.

In the casting and molding process to create the implant, silicone rubber was used to make the mold to capture geometric details. We had chosen bone cement to make hand-crafted skull patches for more than 16 years and found the material satisfactory. Other biocompatible materials^1^ can also be molded to match the shape of the defect in the same way.

The main contributions of this manuscript can be summarized as:

1. We propose an effective deep-learning-based 3D inpainting solution to meet the requirements of cranioplasty.
2. Little or no post-processing is required to eliminate noise in the implant geometry model generated by the proposed approach.
3. The proposed system is computationally efficient and only requires a desktop PC equipped with a GPU accelerated graphics card to perform calculations.

## Results

### Numerical study

This section uses simulation cases to investigate the quantitative performance of the proposed framework.

**Figure 2:**
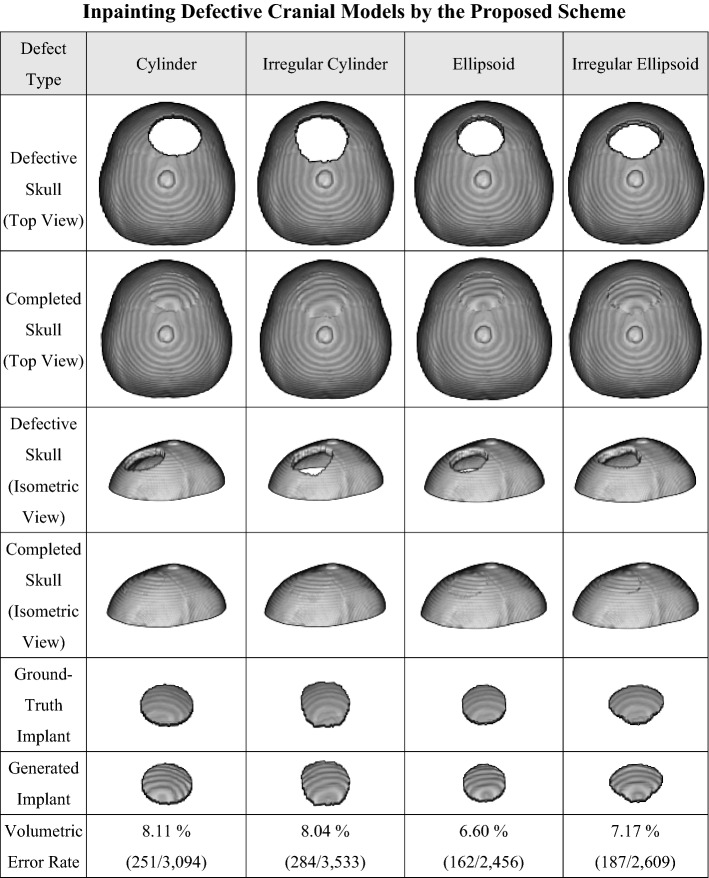
Automatic generation of implants for defective cranial models by the proposed deep learning system. The defects are made by Boolean subtraction of 4 types of 3D masks from an intact skull model.

Figure 2 demonstrates four automatic cranial implant design cases. The upper parts of the defective skulls are displayed in the top view and isometric view in the first and third rows, respectively. The second and fourth rows present the complete skulls generated by the proposed system. The ideal (ground-truth) implants and the created implants are shown in the fifth and sixth rows, respectively.

In the numerical evaluation, we created regular and irregular holes on intact 3D cranium models. The difference between cylindrical and ellipsoidal defects lies in the boundary of the defects. The former is parallel to the axial direction, while the latter is curved, as shown in the flawed skulls of Fig. 2. Please note that all defects pass through the central plane in these cases and therefore cannot be created based on the traditional symmetry assumption.

The implants in the sixth row are obtained by subtracting the original flaw skull models from the generated complete models. If a generated implant is denoted as P* and its corresponding ideal one is expressed as *P*, the volumetric error rate, denoted as *r*, is defined as

1 $$ r\,{ = }\,\frac{{\left\| {P - P^{*} } \right\|_{1} }}{{\left\| P \right\|_{1} }} \cdot 100\,\% $$

where the 1-norm is used. The last row of Fig. 2 quantitatively summarizes the repair performance of the proposed scheme. We can find that the proposed deep learning system achieves a volumetric error rate of less than 8.2% in this case study.

In addition, to understand the limitations of the repair ability of the proposed scheme, we created defects of various sizes and positions on the skull model for numerical study. According to the numerical investigation, detailed in the Supplementary Material, the system can produce satisfactory implants for defects up to 35% in volume.

### Surgical implementation

The proposed deep learning system has been used in implant generation for clinical applications. This section describes one of these successful implementations.

**Figure 3:**
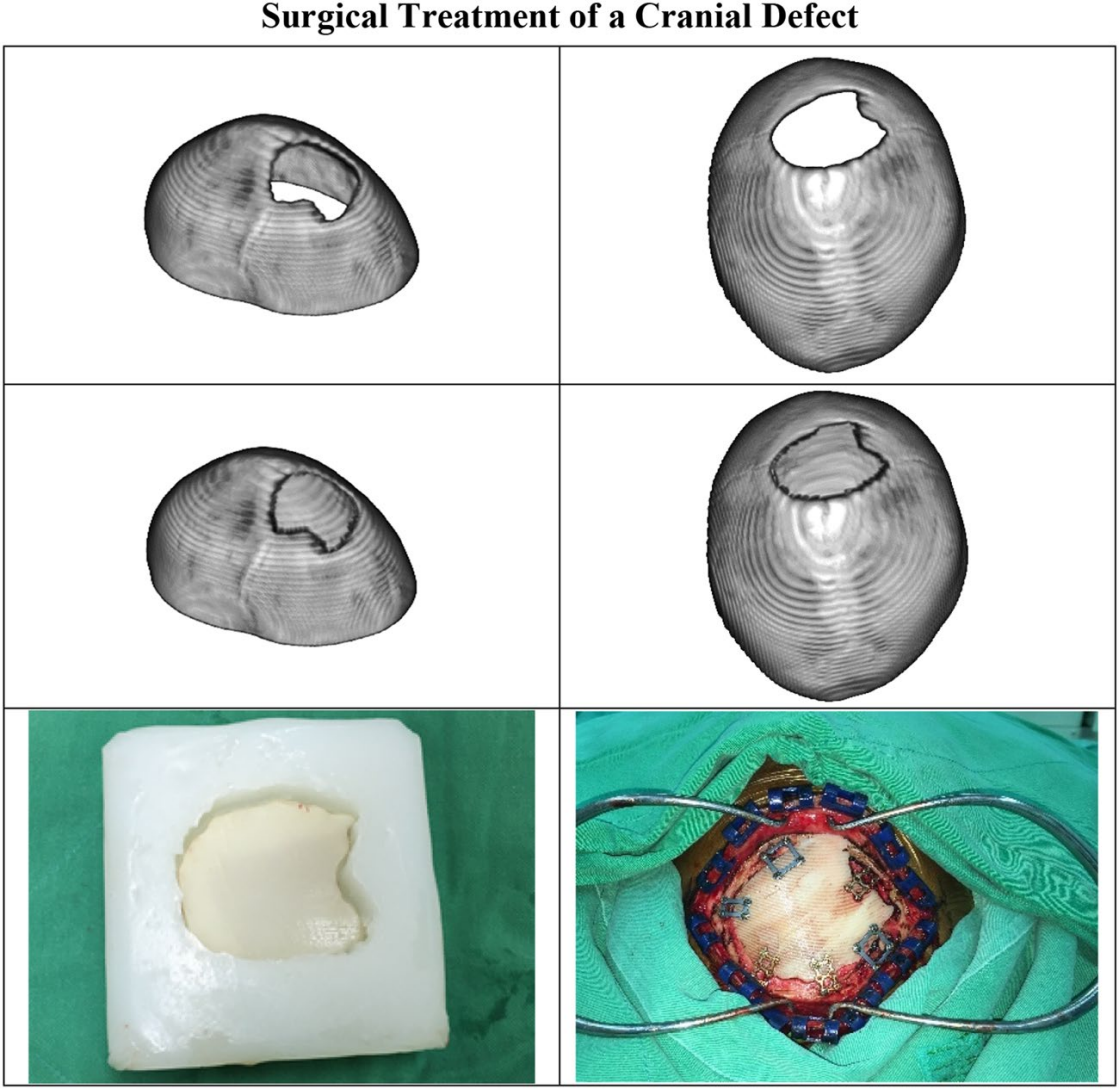
Clinical cranial reconstruction. The first row shows the isometric and top views of the original cranial model. The second row shows the isometric and top views of the repaired cranial model using the implant created by the proposed deep learning system. The last row shows the bone-cement implant molded with silicone rubber (left photo), and a scene of the surgery when the implant was fixed to the skull (right photo).

A 12-year-old boy with a congenital craniofacial defect sought surgical treatment. Computed tomography showed that the longest crack in his sagittal suture was 124 mm in diameter. As shown in Fig. 3, the proposed deep learning system generated an adequate 3D geometry of the implant required to repair the defect.

It is worth mentioning that although the system has been trained on simplified cylindrical and elliptical defects, the geometry of the generated implant is satisfactory for actual implants with irregular boundaries.

## Discussion

Skull implant design usually requires time-consuming human–computer interaction and requires expertise in the medical field. The motivation for this work is the need to automate this process and improve the quality of medical care. We proposed a 3D deep learning network to automatically complete defect models in this study.

Several state-of-the-art deep learning models have achieved great success in the field of computer vision. However, these 2D results cannot be directly extended to 3D problems. For example, the stable training of GANs is more challenging for 3D imaging tasks involving more spatial features.

The performance of the proposed neural network was investigated in both simulated and clinical cases to verify its applicability. According to the numerical study, the proposed deep learning system achieves a volumetric error rate of less than 8.2%. Furthermore, the system can produce satisfactory implants for defects up to 35% in volume. Surgical implementation also showed that the geometry of the resulting implant was satisfactory for actual implants with irregular boundaries.

The capability of the proposed network is made possible through its concise and effective architecture and training methods. The network effectively integrates twelve 3D convolutional layers into a skip-connected autoencoder structure, which includes four dilated convolutional layers. We did not introduce the drop-out mechanism in the network, nor did we introduce batch normalization.

Effective and well-organized training data is also essential for efficient training on such a high-resolution 3D problem. The network inputs are defective 3D models, and the target outputs are the corresponding intact models. Training is efficient because it is based on supervised learning, rather than relying on indirect information, such as the feedback signal provided by the discriminator in the GANs^12^ scheme. The proposed network only requires a graphics card-enhanced desktop PC to compute, which makes the system a vast potential in many clinical applications.

There are several limitations of the proposed approach, however. First, 7154 sets of skull models for the deep learning system training were created through the data augmentation technology from 73 skull models. Although case studies strongly support this approach, further clinical trials are needed to evaluate its feasibility for diverse patients. Second, due to the shortage of computing resources, the repairable area is limited to the upper part of the cranium with a volumetric resolution of 112 × 112 × 40. For clinical practice, several post-processing procedures, including resampling and smoothing, are required to provide a smooth implant model for 3D printing.

Regarding these limitations, this study is a preliminary work, and we believe that it can provide incentive for future advanced research. Future work can focus on increasing the number of skull models, combined with appropriate data enhancement technology and network architecture arrangements, to improve the training quality of the system, and increase the volumetric resolution to 448 × 448 × 160. It is also possible to conduct further studies on skull defects of different sizes and positions, such as the cheekbones and temporal bone regions, to reduce the limitation on the system's repair capabilities.

## Methods

### Cranial dataset

The dataset used for this study is the DICOM (Digital Imaging and Communications in Medicine) metadata collected in the Department of Neurosurgery, Chang Gung Memorial Hospital, Taoyuan, Taiwan. Being authorized by the Institutional Review Board with IRB No. 201900991B0 and Clinical trial/research Consent No. 201801697B0C601, any protected health information was removed from the DICOM metadata.

Each computed tomography (CT) image is with a resolution of 512 × 512 pixels, but the interval between the images can be 0.3 mm, 0.435 mm, 0.5 mm, 0.8 mm, 1.0 mm, 1.25 mm, or 3.0 mm. CT data contains bones and other tissues, and each patient’s taking conditions are different. We set the intensity threshold in the interval of [1200, 1817] according to the Hounsfield unit^27^ to preserve the bone tissue in the data.

Also, the number of parameters in the network is proportional to the complexity of the inpainting task. Therefore, enough examples, at least thousands of data sets, are needed to train the network. Unfortunately, after sifting through 327 sets of collected data, only 73 sets are usable, because many of them are incomplete or applied with bone screws. Hence, we rotate, tilt, and vertically translate the 3D medical images, resulting in 73 × 7 × 7 × 2 = 7154 sets of augmented data^28^. The operations are with intervals of 2 degrees for the rotation and tilting, each with 7 alternatives, and 2 voxels for translation.

Due to calculation efficiency considerations, down-sampling is usually required. The original resolution of all collected DICOM metadata on the XY plane is 512 × 512 pixels. After weighing the conflict between modeling quality and calculation requirements, we have that at least a 112 × 112 plane resolution should be maintained. To further alleviate the computational burden, we only cropped the upper part of the skull models, resulting in normalized datasets with a volumetric resolution of 112 × 112 × 40.

### The proposed 3D deep learning network

Although 2D image completion technology has made significant progress recently, 3D shape processing involves higher dimensions and is still very challenging. Considering that the human skulls have a similar topology, we manage the system to be trained through supervised learning.

In each pair of training sample, the input to the network is a flawed 3D cranial model with a volumetric resolution of 112 × 112 × 40, and the output is the corresponding intact model.

The system is basically a high-dimensional autoencoder^9–11^ augmented with skip-connections. Because of its shape, this architecture is also called U-Net^6^ or V-Net^29^. The autoencoder architecture contains two parts, the encoder and the decoder. The basic autoencoder is dedicated to compressing or reducing information to lower dimensions, denoted as the latent space, in the encoder part, and restored in the decoder part. It has been a mature backbone of many generative tasks^5–12^.

The encoder part of the proposed scheme contains three 3D convolution layers, each is equipped with the Rectified Linear Unit (ReLU)^30^ and is succeeded with a maximum pooling (or max pooling) layer. This part reduces the data size initially to the bottleneck, also known as latent space.

Between the encoder and decoder parts, we use four layers of 3D dilated convolutional layers^31,32^ instead of fully connected layers. Dilated convolution introduces spacings between input values called dilation rate in the kernel of the convolutional layer. For example, in the 3D dilated convolution, a 3 × 3 × 3 kernel with a dilation rate of 2 has the same field of view as a 5 × 5 × 5 kernel, using only 27 parameters. Besides, each dilation layer is equipped with the ReLU activation function.

The use of dilated convolution provides a wide field of view while avoiding multiple convolutions or larger kernels. In other words, the dilation mechanism supports expansion of the receptive field without increasing the number of kernel parameters. These 3D dilated convolutional layers are important for collecting more structural information surrounding the missing parts to generate the patch geometry.

The decoder part contains four 3D convolution layers, each is equipped with the ReLU activation function except the last layer and is succeeded with an up-sampling layer to expand the output to higher resolution. The last layer is equipped with a sigmoid function to normalize the output to the range [0, 1].

There are 8 skip-connections^33^ in the network between the corresponding encoder and decoder layers, and between the neighboring mid-layers. The skip-connections help to enhance the prediction ability of the decoding process and prevent the gradient vanishing in the deep neural network. This structure is similar to the scheme described by Devalla et al.^34^, which is a dilated-residual U-Net for 2D medical image segmentation.

**Figure 4:**
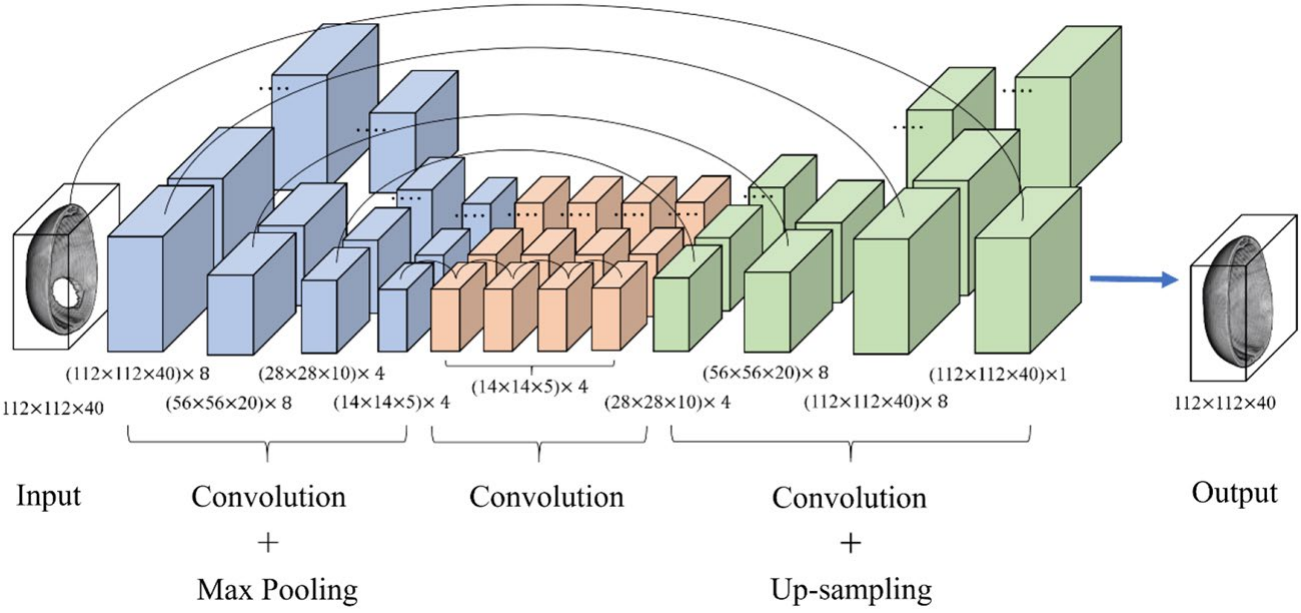
Architecture of the proposed deep learning network.

In summary, the deep learning system consists of twelve 3D convolutional layers, including four 3D expansion layers, three max-pooling layers, three up-sampling layers and eight skip connections. Table 1 and Fig. 4 give an overview of its architecture. The architecture forms a network with a total of 8269 trainable parameters. This concise neural network model is realized by reducing the number of kernels in the convolutional layers to its performance limit.

**Table 1:** Architecture summary of the 3D inpainting network.

Type	Kernel	Dilation	Stride	Channels
3D convolution	3 × 3 × 3	1	1 × 1 × 1	8
3D convolution	3 × 3 × 3	1	1 × 1 × 1	8
3D convolution	3 × 3 × 3	1	1 × 1 × 1	4
3D convolution	3 × 3 × 3	1	1 × 1 × 1	4
Dilated 3D convolution	3 × 3 × 3	2	1 × 1 × 1	4
Dilated 3D convolution	3 × 3 × 3	4	1 × 1 × 1	4
Dilated 3D convolution	3 × 3 × 3	8	1 × 1 × 1	4
Dilated 3D convolution	3 × 3 × 3	16	1 × 1 × 1	4
3D deconvolution	3 × 3 × 3	1	1 × 1 × 1	4
3D deconvolution	3 × 3 × 3	1	1 × 1 × 1	8
3D deconvolution	3 × 3 × 3	1	1 × 1 × 1	8
Output	3 × 3 × 3	1	1 × 1 × 1	1

“Channels” refers to the number of kernels for the corresponding layer.

**Figure 5:**
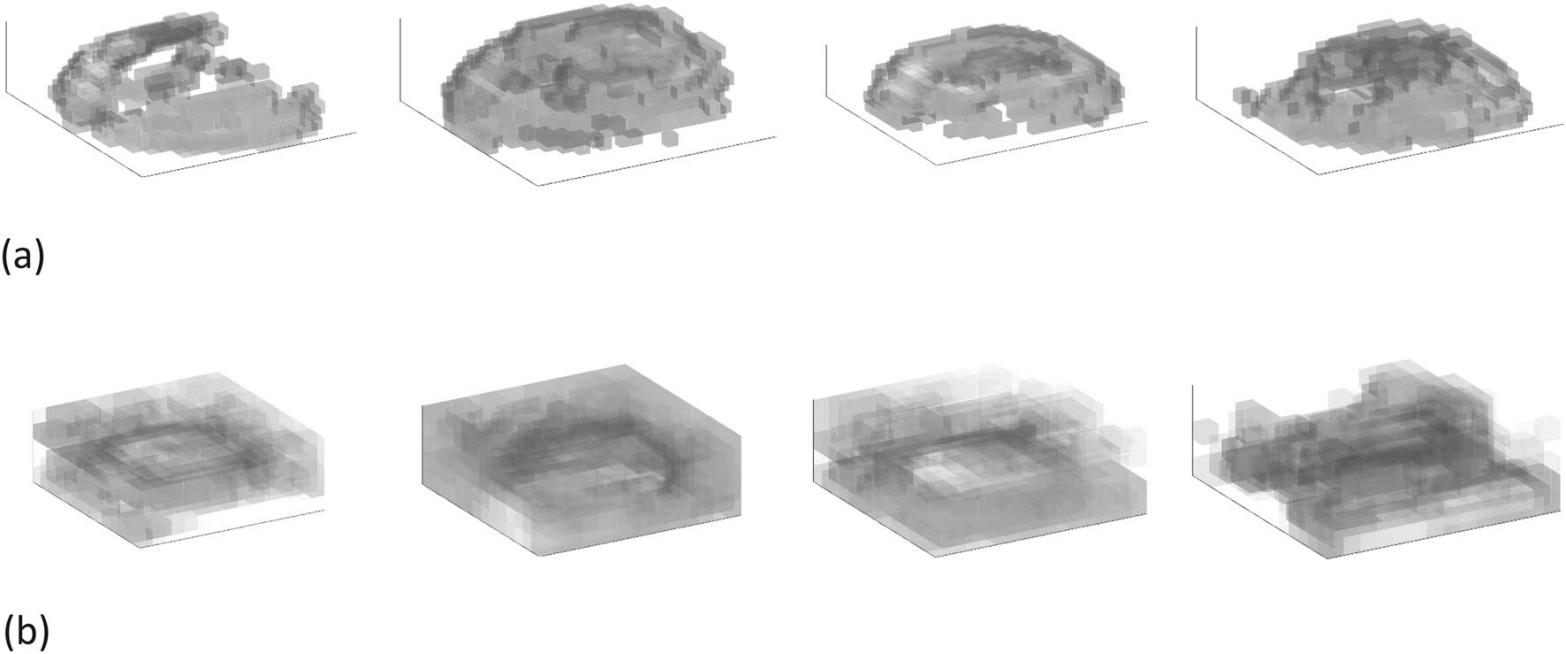
Visualization of typical 3D feature maps before and after the four dilation layers. Detailed input and output datasets are presented in the Supplementary Material. (**a**) The 3D feature maps generated after the encoder and before the dilation layers. Each map is with a volumetric resolution of 28 × 28 × 10. (**b**) The 3D feature maps generated after the four dilation layers and before the decoder layers. Each map is with a volumetric resolution of 14 × 14 × 5.

We can visualize the internal data corresponding to a specific input model to explore the computational behavior of the deep learning system. In 3D convolutions, kernels can move in 3 directions and thus the feature maps obtained are also 3D. Figure 5 shows the 3D feature maps generated before and after the 4 dilated convolutional layers. Note that there are 4 dilated convolutional layers in the system, and each layer is equipped with 4 kernels. We can see from Fig. 5 that, as the data is processed along the layers, the defective region reduces its size.

The input required to create the 3D feature maps of Fig. 5 are described in the Supplementary Material. Diagrams of kernels and more feature maps are also presented in it for further investigation.

**Figure 6:**
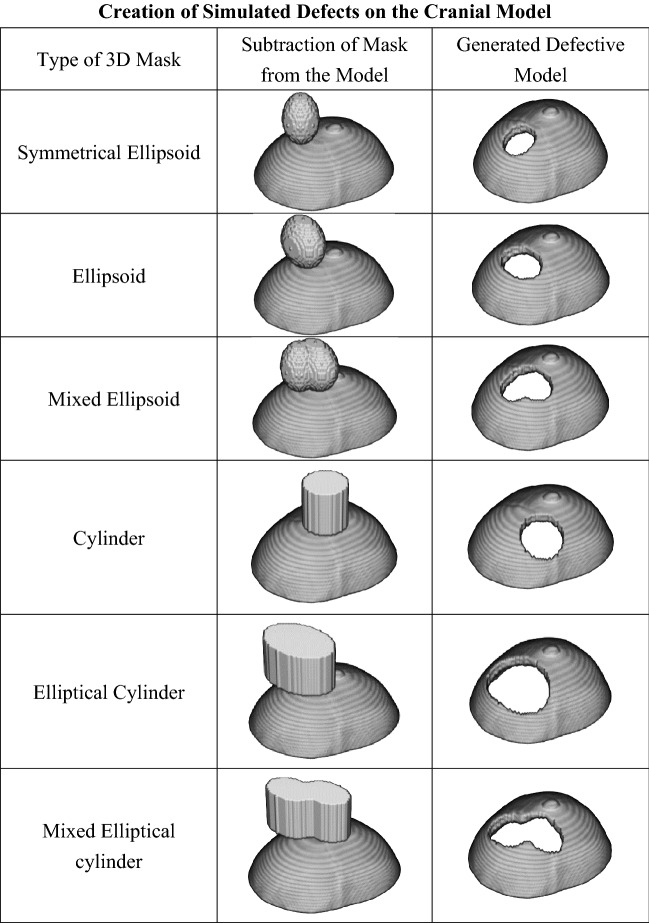
Various types of 3D masks are used to generate defects on the cranial model to train the proposed deep learning system.

The data size of each 112 × 112 × 40 skull model is 2 MB, and the 7150 training sets amount to 14.35 GB. To provide defective skull models for training, we randomly apply six types of 3D masks with equal probability: symmetrical ellipsoid, ellipsoid, mixed ellipsoid, cylinder, elliptical cylinder, and mixed elliptical cylinder, as shown in Fig. 6.

In training the network, a batch size of 10 models was applied, and we used Adadelta^35^ as the optimizer and Binary Cross entropy as the cost function. Adadelta^35^ is an extension of Adagrad^36^, which can dynamically adjust learning rate over time without setting parameters. The main difference between these two optimizers is that Adagrad accumulates all previous gradient squares, while Adadelta only accumulates a fixed number of values. The same settings were applied to train and evaluate our model. The enhanced data is randomly divided into a training set and a validation set, and the validation split is 0.1. In other words, the validation set is 10% of the available data.

The time required for a training session of 1200 epochs took 58.4 h. Once trained, a completion task takes only 8.6 s. Details of the computational settings and the training history of the proposed deep learning model are provided in the Supplementary Material.

## Supplementary Information

Supplementary Information.

## Data Availability

*Database* The DICOM data set used in this study was collected in the Department of Neurosurgery, Chang Gung Memorial Hospital, Taoyuan, Taiwan from 2012 to 2021. Being authorized by the Institutional Review Board with IRB No. 201900991B0 and Clinical trial/research Consent No. 201801697B0C601, any protected health information was removed from the DICOM metadata. *Software* All the images shown in this article were created using MathWorks' MATLAB^®^ 2020b, and the graphic of Fig. 4 were created using Microsoft Office 365.
