# Supplementary material for: Three-dimensional deep learning to automatically generate cranial implant geometry
Source: Sci Rep. 2022 Feb 17;12:2683. doi: 10.1038/s41598-022-06606-9 (PMC8854612; doi:10.1038/s41598-022-06606-9)
Supplement: Supplementary file 1 — Supplementary Information. [file 41598_2022_6606_MOESM1_ESM.docx]

**Three-Dimensional deep learning to automatically generate cranial implant geometry**

Chieh-Tsai Wu, Yao-Hung Yang & Yau-Zen Chang

**Supplementary Material**

Our deep learning system were trained on a desktop PC with the following specifications:

1. Python 3.7.7
2. TensorFlow 2.1.0
3. Keras 2.3.1
4. CUDNN 7.6.5
5. CUDA 10.1.243
6. Intel Core i9 9900K CPU @3.60GHz
7. One nVIDIA GeForce RTX 2080 Ti graphics card
8. 32GB DDR4 memory
9. Windows 10: 64-bit

The detailed architecture of the proposed deep learning model is shown in Fig. S1, and the training history is shown in Fig. S2, where the validation split is 0.1. The time required for a training session of 1,200 epochs took 58.4 hours. We use the same settings to train and evaluate our model. Once trained, a completion task takes only 4.6 seconds.

-----------------------------------------------------------------------------------------

Layer (type) Output Shape Param # Connected to

**====================================================**

input_1 (InputLayer) (None, 112, 112, 40, 1) 0

-----------------------------------------------------------------------------------------

conv3d_1 (Conv3D) (None, 112, 112, 40, 8) 224 input_1[0][0]

-----------------------------------------------------------------------------------------

conv3d_2 (Conv3D) (None, 112, 112, 40, 8) 1,736 conv3d_1[0][0]

-----------------------------------------------------------------------------------------

max_pooling3d_1 (MaxPooling3D) (None, 56, 56, 20, 8) 0 conv3d_2[0][0]

-----------------------------------------------------------------------------------------

conv3d_3 (Conv3D) (None, 56, 56, 20, 4) 868 max_pooling3d_1[0][0]

-----------------------------------------------------------------------------------------

max_pooling3d_2 (MaxPooling3D) (None, 28, 28, 10, 4) 0 conv3d_3[0][0]

-----------------------------------------------------------------------------------------

conv3d_4 (Conv3D) (None, 28, 28, 10, 4) 436 max_pooling3d_2[0][0]

----------------------------------------------------------------------------------------max_pooling3d_3 (MaxPooling3D) (None, 14, 14, 5, 4) 0 conv3d_4[0][0]

----------------------------------------------------------------------------------------conv3d_5 (Conv3D) (None, 14, 14, 5, 4) 436 max_pooling3d_3[0][0]

-----------------------------------------------------------------------------------------

add_1 (Add) (None, 14, 14, 5, 4) 0 max_pooling3d_3[0][0]

conv3d_5[0][0]

-----------------------------------------------------------------------------------------

conv3d_6 (Conv3D) (None, 14, 14, 5, 4) 436 add_1[0][0]

-----------------------------------------------------------------------------------------

add_2 (Add) (None, 14, 14, 5, 4) 0 conv3d_5[0][0]

conv3d_6[0][0]

-----------------------------------------------------------------------------------------

conv3d_7 (Conv3D) (None, 14, 14, 5, 4) 436 add_2[0][0]

-----------------------------------------------------------------------------------------

add_3 (Add) (None, 14, 14, 5, 4) 0 conv3d_6[0][0]

conv3d_7[0][0]

-----------------------------------------------------------------------------------------

conv3d_8 (Conv3D) (None, 14, 14, 5, 4) 436 add_3[0][0]

-----------------------------------------------------------------------------------------

add_4 (Add) (None, 14, 14, 5, 4) 0 conv3d_7[0][0]

conv3d_8[0][0]

-----------------------------------------------------------------------------------------

conv3d_9 (Conv3D) (None, 14, 14, 5, 4) 436 add_4[0][0]

-----------------------------------------------------------------------------------------

up_sampling3d_1 (UpSampling3D) (None, 28, 28, 10, 4) 0 conv3d_9[0][0]

-----------------------------------------------------------------------------------------

add_5 (Add) (None, 28, 28, 10, 4) 0 max_pooling3d_2[0][0]

up_sampling3d_1[0][0]

-----------------------------------------------------------------------------------------

conv3d_10 (Conv3D) (None, 28, 28, 10, 8) 872 add_5[0][0]

-----------------------------------------------------------------------------------------

up_sampling3d_2 (UpSampling3D) (None, 56, 56, 20, 8) 0 conv3d_10[0][0]

-----------------------------------------------------------------------------------------

add_6 (Add) (None, 56, 56, 20, 8) 0 max_pooling3d_1[0][0]

up_sampling3d_2[0][0]

-----------------------------------------------------------------------------------------

conv3d_11 (Conv3D) (None, 56, 56, 20, 8) 1,736 add_6[0][0]

-----------------------------------------------------------------------------------------

up_sampling3d_3 (UpSampling3D) (None, 112, 112, 40, 8) 0 conv3d_11[0][0]

-----------------------------------------------------------------------------------------

add_7 (Add) (None, 112, 112, 40, 8) 0 conv3d_1[0][0]

up_sampling3d_3[0][0]

-----------------------------------------------------------------------------------------

conv3d_12 (Conv3D) (None, 112, 112, 40, 1) 217 add_7[0][0]

-----------------------------------------------------------------------------------------

add_8 (Add) (None, 112, 112, 40, 1) 0 input_1[0][0]

conv3d_12[0][0]

**====================================================**

Total params: 8,269

Trainable params: 8,269

Non-trainable params: 0

**Figure S1.** Architecture of the proposed deep learning model.
(Created by the summary method of Keras.)


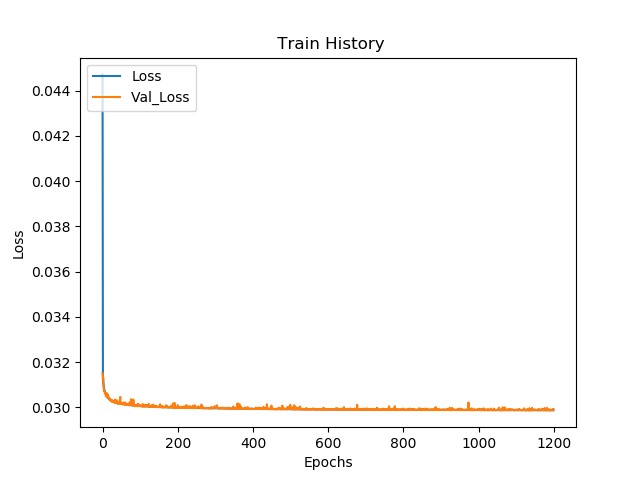


**(a)** The overall 1,200 epochs.


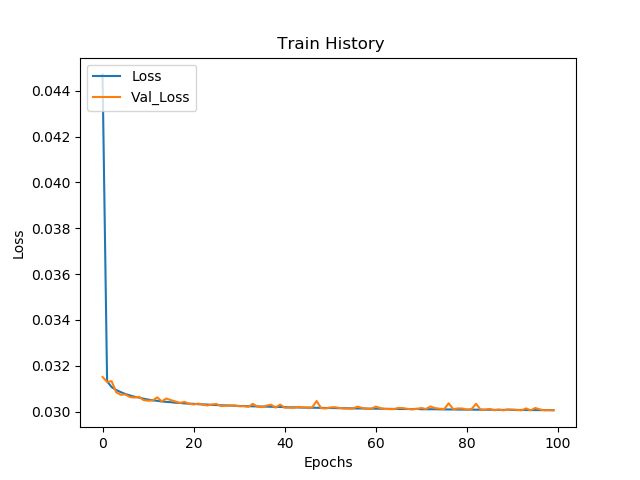


**(b)** The first 100 epochs of (a).

**Figure S2.** Training history of the proposed deep learning model.

Due to efficiency issues in the calculation, down-sampling is usually required. The original resolution of all collected DICOM data is 512×512 pixels. Figure S2 shows a 3D model in various resolutions. After weighing the conflict between modeling quality and calculation requirements, we have that 76×76 is almost the lower limit of 3D modeling quality, and 112×112 is the resolution that should be maintained at least.


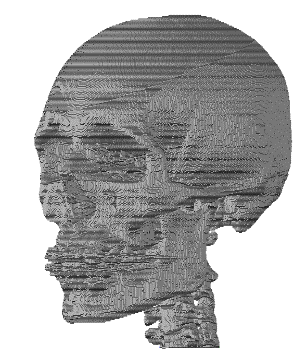

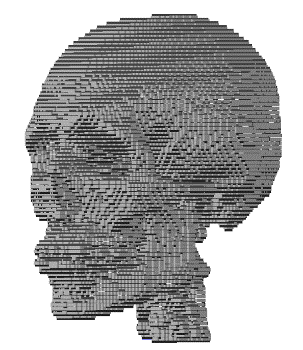

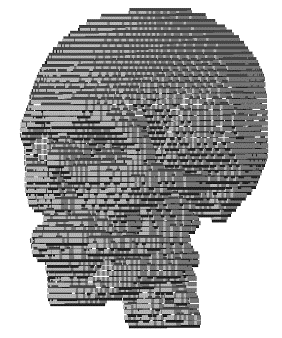


(a) (b) (c)

**Figure S3.** 3D skull models in various resolutions downgraded from the original resolution of 512 × 512 × 396. (a) 190 × 190 × 147, Voxel Size: 0.980469 × 0.980469 × 1.6, (b) 112 × 112 × 89, Voxel Size: 1.63411 × 1.63411 × 2.6667, (c) 76 × 76 × 59, Voxel Size: 2.45117 × 2.45117 × 4.

The visualization of weights and feature maps provides a deeper understanding of the proposed deep learning system. We can check the kernels learned by the system to investigate the types of features to be detected.

As shown in Figure S4, we could visualize all the kernels in the first 3D convolution layer of the deep learning system as eight 3×3×3 cubes, one for each kernel. Dark voxels represent small or suppression weights, while light squares represent large or excitement weights. We have that the kernels are different from each other.

The deep learning system consists of twelve 3D convolutional layers, and all 65 kernels can be visualized in the same way.

| 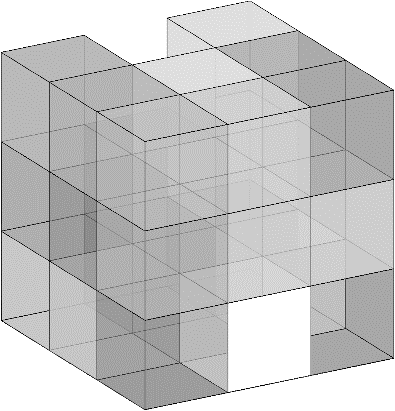 | 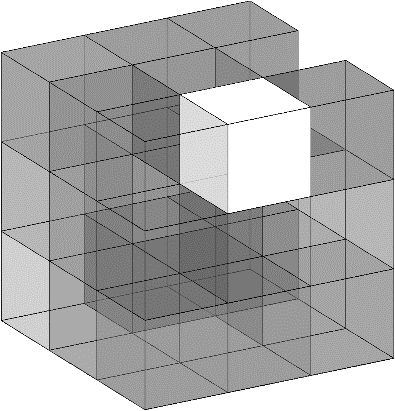 | 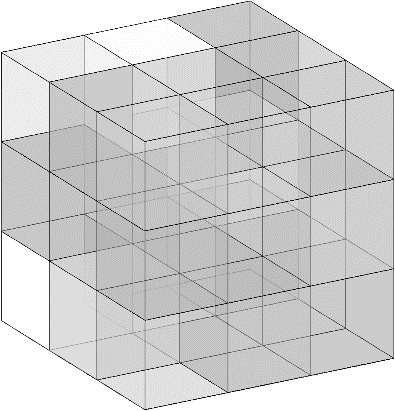 | 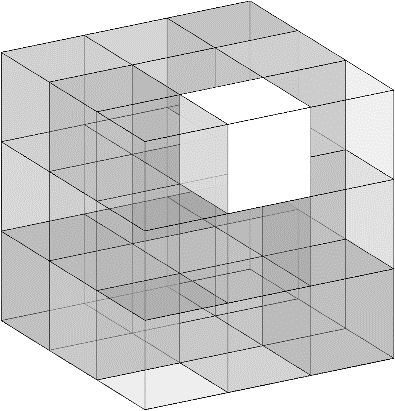 |
| --- | --- | --- | --- |
| 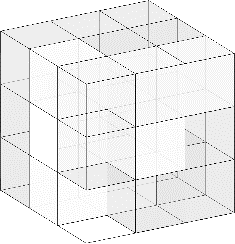 | 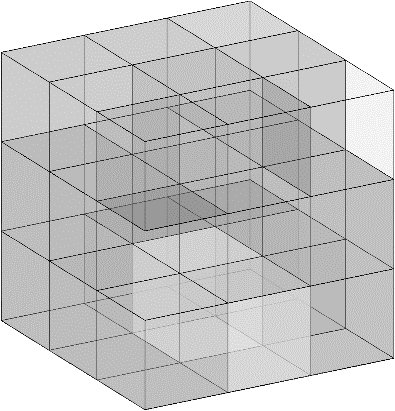 | 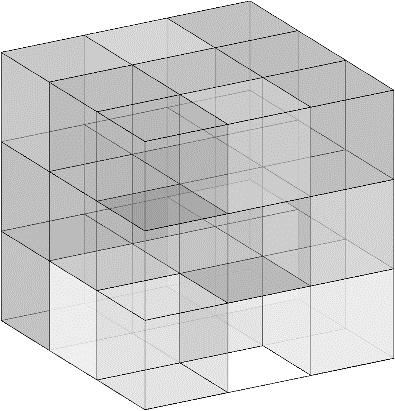 | 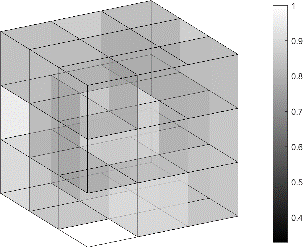 |

**Figure S4.** The 8 kernels in the first convolutional layer of the proposed network, denoted as conv3d_1 in Figure S1.

In order to explore the visualization of feature maps, we need input for the deep learning system to create activations. The following example is created by a defective model shown in Figure S5 (a). Its corresponding model completed by the deep learning system is given in Figure S5 (b).

We can check the feature maps generated by the convolutional layers. The following figures, Figure S6 and S7, illustrate how the proposed network works in some of the deep learning layers.

| 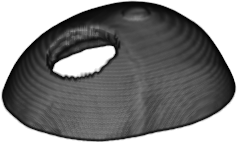 | 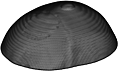 |
| --- | --- |
| (a) Defective Model | (b) Generated Model |

**Figure S5.** Example model for the investigation of kernels and 3D feature maps. (a) 3D defective skull model. (b) Completed model generated by the proposed deep learning system. These two models are with a volumetric resolution of 112 × 112 × 40.

| 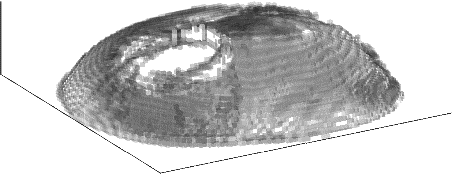 | 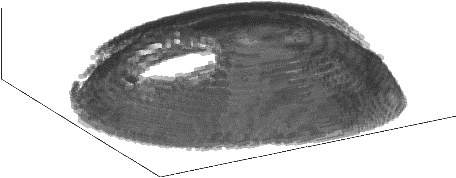 | 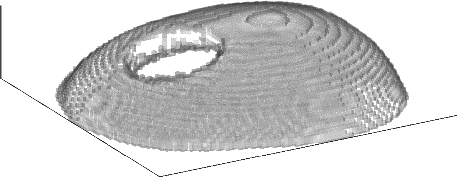 | 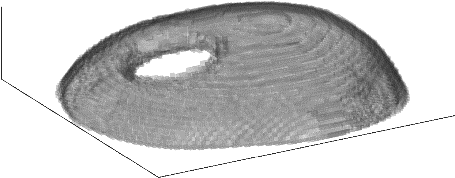 |
| --- | --- | --- | --- |
| 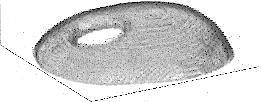 | 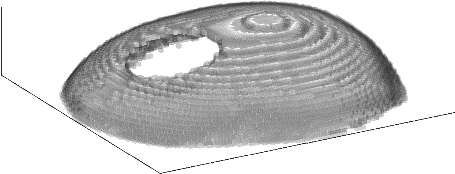 | 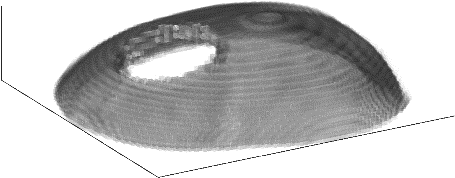 | 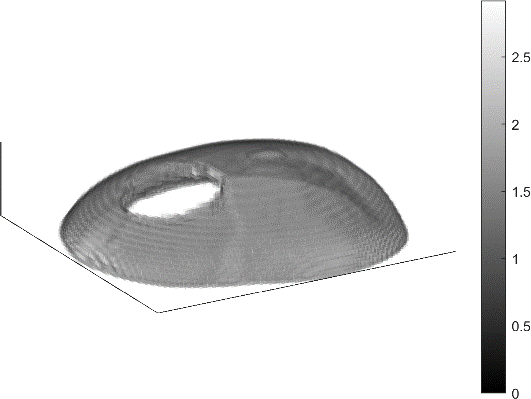 |

**Figure S6.** Feature maps generated by the first 3D convolutional layer of the proposed deep learning system, denoted as conv3d_1 in Figure S1. These 3D feature maps are with a volumetric resolution of 112 × 112 × 40.

| 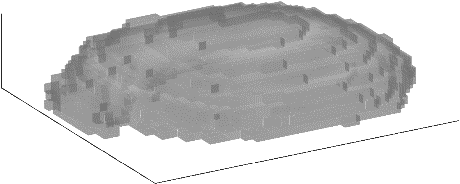 | 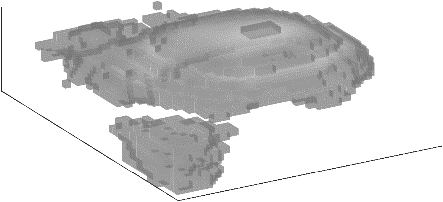 | 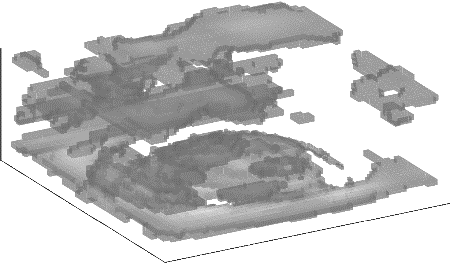 | 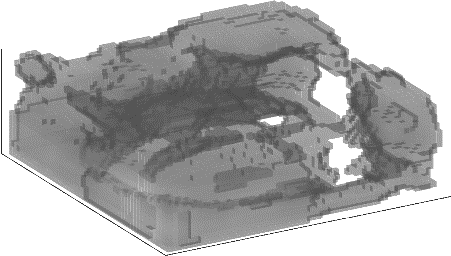 |
| --- | --- | --- | --- |
| 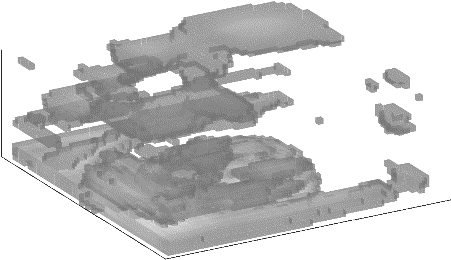 | 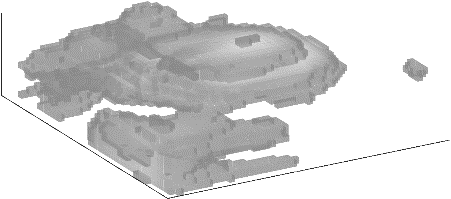 | 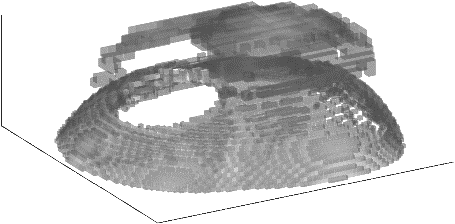 | 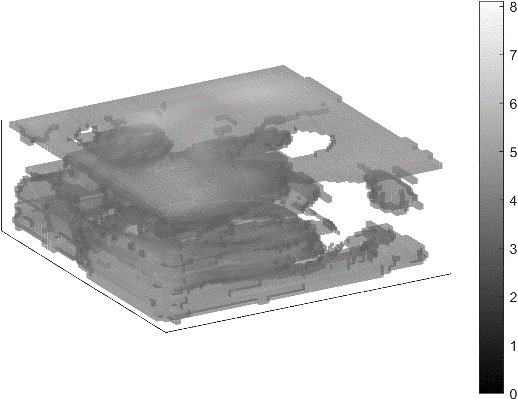 |

**Figure S7.** Feature maps generated by the last 3D up-sampling layer of the proposed deep learning system, denoted as conv3d_11 in Figure S1. These 3D feature maps are with a volumetric resolution of 112 × 112 × 40.

There are several publicly available skull datasets. We selected three cases from them to give our results an opportunity to compare with other studies. Each of the original skull model has a different resolution. Before conducting the task, we resampled the models and normalized them to a volume resolution of 112×112×40, as shown in Fig. S8.

1. Case 1.
   The original data is 1.953124 mm × 1.953124 mm × 2.50 mm, gray scale.
   Selected from #117 skull model of the CQ500 Dataset.
   Downloadable from the website: http://headctstudy.qure.ai/dataset.
2. Case 2.
   The original data is 1.804688 mm × 1.804688 mm × 2.531992 mm, binary.
   Selected from #000 skull model of the the AutoImplant 2020 Example (the MICCAI 2020 Cranial Implant Design Challenge).
   Downloadable from the website: https://autoimplant.grand-challenge.org/Home/.
3. Case 3.
   The original data is 1.6 mm × 1.6 mm × 1.6 mm, binary.
   Selected from #000 skull model of the the AutoImplant 2021 Example (2nd MICCAI challenge on cranial implant design).
   Downloadable from the website: https://autoimplant2021.grand-challenge.org/.

| Cases | Original Model | Normalized Model |
| --- | --- | --- |
| Case 1 | 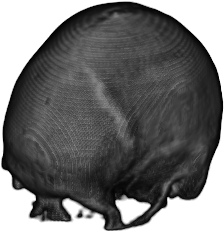 | 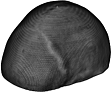 |
| Case 2 | 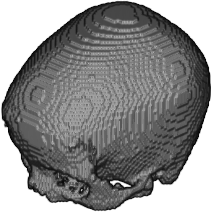 | 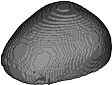 |
| Case 3 | 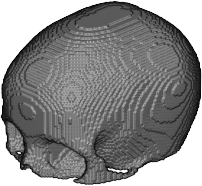 | 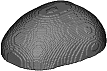 |

**Figure S8.** Cranial models selected from three publicly available datasets for performance comparison.

As shown in Fig. S9, the volume error rates of Case 2 and Case 3 are higher than Case 1. The volumetric error rate of Case 1, 8.14 %, is similar to the cases shown in Fig. 2. This is because our proposed network is trained with grayscale data, while the datasets of Case 2 and Case 3 are binary.

Nonetheless, all the patch models are noise-free and suitable for creating patches.

| Case | Defective  Cranial Model | Completed Cranial Model | Ideal Implant | Generated  Implant | Volumetric  Error Rate |
| --- | --- | --- | --- | --- | --- |
| Case 1 | 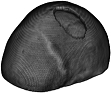 | 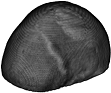 | 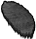 | 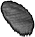 | 8.14 % (152/1,868) |
| Case 2 | 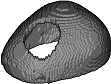 | 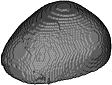 | 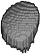 | 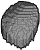 | 14.91 % (488/3,273) |
| Case 3 | 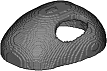 | 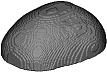 | 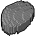 | 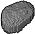 | 15.16 % (465/3,068) |

**Figure S9.** The repair performance of our proposed method when applied to the three cases shown in Figure S8.

| **Repair Performance Limit of the Proposed System**  **(Top View)** | | |
| --- | --- | --- |
| **Volumetric Percentage** | **Defect Skull** | **Output of the Proposed Scheme** |
| 28.72 %  ( 7,757 / 27,007 ) |  |  |
| 38.99 %  ( 10,530 / 27,007 ) |  |  |
| 42.64 %  ( 11,517 / 27,007 ) |  |  |
| 38.07 %  ( 10,282 / 27,007 ) |  |  |
| 42.51 %  (11,482 / 27,007 ) |  |  |
| 47.03 %  ( 12,701 / 27,007 ) |  |  |

In order to understand the limitations of the repair capabilities of the proposed scheme, we created defects of various sizes and positions on the skull model. According to numerical simulations, the system can produce satisfactory repair effects for defects up to 35 % by volume.

**Figure S10.** Repair limit in top view.

| **Repair Performance Limit of the Proposed System**  **(Isometric View)** | | |
| --- | --- | --- |
| **Volumetric Percentage** | **Defect Skull** | **Output of the Proposed Scheme** |
| 28.72 %  ( 7,757 / 27,007 ) |  |  |
| 38.99 %  ( 10,530 / 27,007 ) |  |  |
| 42.64 %  ( 11,517 / 27,007 ) |  |  |
| 38.07 %  ( 10,282 / 27,007 ) |  |  |
| 42.51 %  (11,482 / 27,007 ) |  |  |
| 47.03 %  ( 12,701 / 27,007 ) |  |  |

**Figure S11.** Repair limit in isometric view.
